# Multiplexed Photonic Crystal Fiber Gas-Sensing Network Based on Intracavity Absorption

**DOI:** 10.3390/s22239237

**Published:** 2022-11-28

**Authors:** Guangyao Wang, Jianping Sun, Ting Li, Hongjun Wang, Jiahao Li

**Affiliations:** National Institute of Metrology, Beijing 100029, China

**Keywords:** gas sensing network, intracavity absorption, hollow core photonic crystal fiber, voltage gradient method

## Abstract

A highly sensitive hollow-core photonic crystal fiber (HC-PCF) gas-sensing network based on intracavity absorption is designed and experimentally verified. The capacity of the multichannel sensing network is expanded by time division multiplexing and wavelength division multiplexing technology. The voltage gradient method is employed in the wavelength scanning process of Fabry–Perot (F-P) filter to enhance the detection efficiency up to six times. The proposed sensing network has 16 sensing points. Experimental results show that the minimum detection limit (MDL) of this sensing system is 25.91 ppm and 26.85 ppm at the acetylene gas absorption peaks of 1530.371 nm and 1531.588 nm, respectively. As far as we know, it is the first time to obtain an intracavity sensing network via the application of an optical switch and DWDM at the same time. The sensing network can be used for high-capacity, low-concentration dangerous gas detection. It has great potential in environmental monitoring, industrial manufacturing, safety inspection and similar occasions.

## 1. Introduction

With the development of industry, air pollution has attracted much attention. The detection of low-concentration gases, such as acetylene, ammonia, carbon monoxide and methane [1,2,3,4], has been attracting the interest of researchers. Optical fiber gas sensors have been widely used in the field of environmental on-line monitoring in the last decades because of their excellent features such as electromagnetic immunity, remote and continuous monitoring, low transmission loss and stability [5,6,7]. Among all kinds of optical fiber gas sensors, the optical fiber gas sensor based on intracavity absorption has the advantages of a high signal-to-noise ratio (SNR), high sensitivity and high resolution, and has become a research hotspot in the gas detection field [8,9,10].

In 1992, an intracavity absorption gas sensor based on an erbium-doped fiber laser was first proposed by Baev V. M. et al. [11]. Then they systematically described and analyzed the principle of the intracavity absorption spectra detection technology, which provided a strong theoretical basis for the intracavity absorption gas sensor. In 2004, Zhang M. et al. proposed an intracavity absorption sensor to detect the mixture of acetylene and nitrogen [12]. The minimum detection limit (MDL) is 10,200 ppm. Then, the wavelength modulation and second harmonic detection are applied to the system, and the MDL is as low as 1000 ppm. In 2011, Liu K. et al. introduced a wavelength modulation technique (WMT) and wavelength sweep technique (WST) into the intracavity absorption optical fiber sensing system to achieve high-sensitivity acetylene gas concentration detection [13]. The experimental results showed that the MDL is 7.5 ppm. In 2016, Yu L. et al. combined the second harmonic with the intracavity absorption spectroscopy technology to realize the concentration detection of acetylene, carbon monoxide and carbon dioxide [14]. The MDL were 0.6 ppm, 17.4 ppm and 19.2 ppm, respectively.

However, most of the previously reported intracavity absorption sensors can only be used to detect a single point. In the actual environment, the detection of gas concentration at a certain point cannot accurately predict the change trend of gas concentration in the environment. With the development of optical communication technology, different kinds of optical gas sensing networks with multi-point on-line detection have gradually developed. At present, the low cost and high efficiency of multi-point intracavity absorption gas sensors are the research hotspots in this field. In 2008, Liu K. et al. achieved multi-point gas concentration measurements by using an optical switch to connect multiple gas cells in the intracavity absorption optical fiber sensor [15]. In 2014, an intracavity absorption multiplexed sensing network based on a dense wavelength division multiplexing (DWDM) filter was designed by Zhang et al. [16]. The MDL was up to 100 ppm at the wavelength of 1536.71 nm. However, due to the use of multiple DWDM filters and couplers, the SNR of each channel was relatively low, which was not conducive to the high-sensitivity detection of output spectra. In 2017, a dual-point automatic switching intracavity-absorption photonic crystal fiber gas sensor based on mode competition was designed by Zhang et al. [17]. The MDL was 398 ppm. In 2018, Zhao et al. proposed an all-fiber gas sensor with intracavity photothermal spectroscopy [18]. A 0.62 m long hollow-core photonic crystal fiber (HC-PCF) was used as the gas cell and the experiment results showed the sensor can realize the measurement with an acetylene concentration of 176 ppm acetylene. Although the multi-point intracavity absorption optical fiber sensing networks could be realized, their cost was relatively high and the commercialization process was limited because of the use of various optical devices, such as a Mach–Zehnder (M-Z) modulator and a photothermal device.

In this paper, a highly sensitive and low-cost intracavity absorption gas sensing network based on erbium-doped fiber ring laser is proposed and demonstrated. An optical switch and DWDM are applied at the same time to realize a 16-channel sensing network. Time division multiplexing (TDM) and wavelength division multiplexing (WDM) technology are applied at the same time, not only expanding the number of sensing channels but also guaranteeing the SNR in each channel. HC-PCF is used as a gas cell to build an all-fiber sensing system, which is a benefit for remote distributed sensing. A voltage gradient method is used to control the tunable Fabry–Perot (F-P) filter, improving the sensing efficiency greatly. Taking the acetylene concentration detection as an example, we chose two absorption peaks at 1530.37 nm and 1531.588 nm to analyze the characteristics of our proposed sensing network, where the acetylene has relatively strong absorption intensity. Experimental results show that our smart sensing network is efficient and has a high sensitivity for the detection of low-concentration acetylene gas. The MDL is 25.91 ppm and 26.85 ppm at the absorption peaks of 1530.371 nm and 1531.588 nm, respectively.

## 2. Experiment Setup and Principle

The working principle of the sensor network is based on intracavity absorption spectroscopy. Analyte with certain absorption characteristics, such as gas or liquid, are placed in the resonant cavity of the ring fiber laser, whose unique absorption spectra can be obtained by scanning the output wavelength of the laser by a tunable F-P filter. When the concentration of the analyte in the resonator changes, the output power at the absorption peaks will change at the same time. Because there is a one-to-one correspondence between the concentration and the absorption intensity of the analyte, the concentration of the analyte can be obtained by detecting the intensity at the absorption peaks. According to the Beer–Lambert law, the concentration of the analyte in the resonant cavity can be calculated by the following formula [19,20]:

(1) $$I_{o}\left(v\right)=I_{i}\left(v\right)e^{-P\cdot S\left(T,v_{0}\right)\cdot g\left(v-v_{0}\right)\cdot L\cdot C}$$

where *I*_o_(*v*) is the intensity of the transmitted light and *I_i_*(*v*) is the intensity of the incident light. *P* is the physical quantity related to the gas pressure. *v* is the incident light frequency and *v*_0_ is the original frequency. *S*(*T*, *v*_0_) is the absorption strength of the analyte at *v*_0_ when the temperature is *T*. *g*(*v*-*v*_0_) is the absorption line of the analyte at *v*. *C* is the concentration of the analyte. *L* is the effective length of the interaction between light and the analyte. Equation (1) demonstrates that the attenuation of the output power *A* = *I_i_*(*v*) − *I*_o_(*v*) caused by the intracavity gas absorption is proportion to the optical path length of the absorber decided by the HCPCF length.

The above formula is an exponential decay function. Therefore, when the concentration of the analyte is low, the light intensity of the output signal can be almost linearly attenuated with the increase in the concentration. However, when the concentration of the analyte is high, the change trend of the light intensity of the output signal will gradually slow down with the increase in the concentration. Thus, when the laser is used to detect low-concentration analyte, the output signal light has a higher power change rate, which will be corresponding to a higher sensitivity. At the same time, the light will pass through the analyte repeatedly in the optical resonant cavity, so the effective length of the interaction between the light and the analyte will increase by several orders of magnitude. According to the Beer–Lambert law, the increase in effective length makes the absorption intensity of analyte increase rapidly, so the intracavity absorption method will greatly improve the detection sensitivity [21].

**Figure 1:**
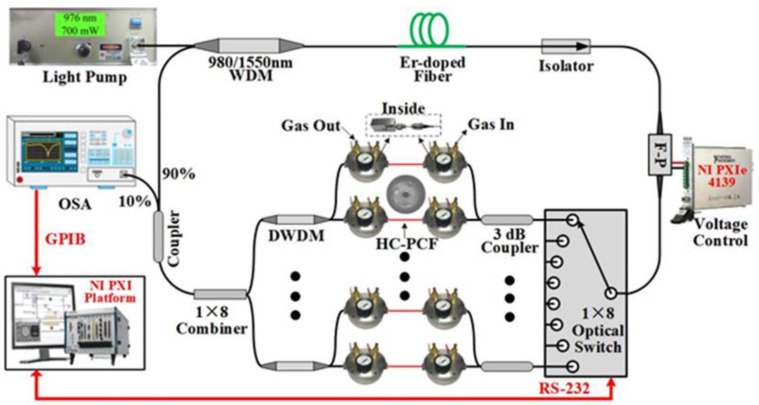
Experimental setup for the intracavity absorption gas sensor network.

In this paper, an intracavity absorption gas sensor network for acetylene gas concentration detection is proposed. The schematic setup of the intracavity absorption gas sensor network is shown in Figure 1. A 976 nm light pump is connected to a filter wavelength division multiplexing (FWDM), pumping a 2 m long erbium-doped fiber (EDF). An optical isolator is inserted between the EDF and a tunable F-P filter to guarantee the ring fiber laser can operate in different wavelength bands. The F-P filter (Micron Optics Inc., Atlanta, GA, USA) with 108 nm free spectral range and fineness of 10,090 is employed to filter the light wave and scan the output wavelength, which is controlled by a precision source measure unit (SMU) with a resolution of 100 nV (PXIe-4139, NI Inc., Austin, TX, USA). To maximize the amount of sensing points, TDM and WDM technology are applied at the same time, which is realized by a 1 × 8 optical switch and eight DWDM filters with the same parameters, respectively. According to the absorption peak of acetylene gas and the International Telecommunication Union (ITU) standard grid channel (100 GHz and 200 GHz spacing), the DWDM filter is chosen to contain one main absorption peak at least within its passband. Through making full use of the DWDM, two sensing points can be distinguished with only one DWDM. Compared with the method of splitting the optical channel using a 3 dB coupler, the DWDM can avoid extra coupling loss. A 1 × 8 combiner is used to combine all the common ports of DWDM filters and then is spliced to a 10:90 output coupler. The 10% port and 90% port are connected to an optical spectra analyzer (OSA) as output laser and FWDM as feedback light, respectively.

**Figure 2:**
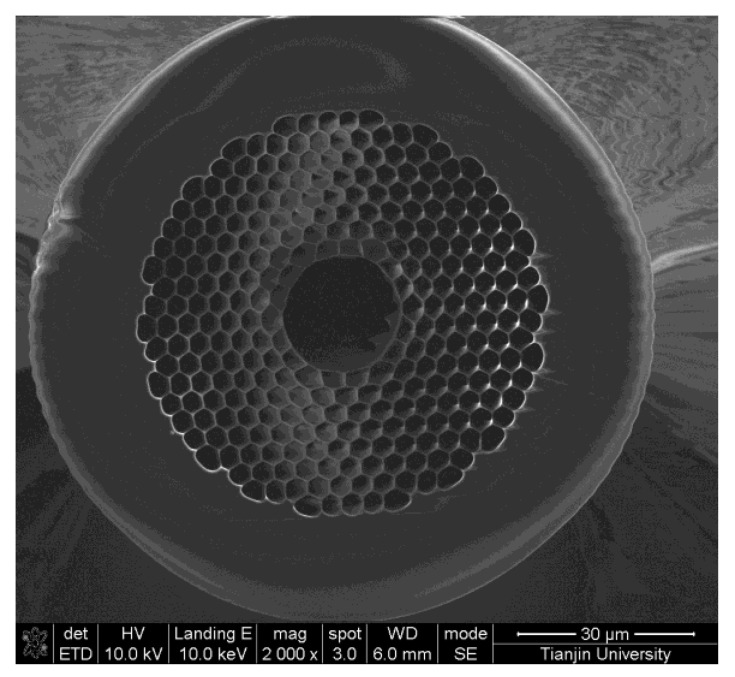
The cross-section of the HC-PCF.

A 1 m long commercially available HC-PCF (HC19-1550, NKT Photonics Inc., Portland, OR, USA) is used to fill acetylene gas. As the cross-section of HC-PCF illustrated in Figure 2, the diameters of core, mode field, holey region and outer cladding are 20 ± 2 μm, 13 ± 3 μm, 70 ± 5 μm and 115 ± 3 μm, respectively. In addition, its attenuation is less than 20 dB/km at the operation wavelength of 1520–1600 nm. As shown in Figure 1, the HC-PCF is connected between a DWDM and a 3 dB coupler through two bare fiber adapters, whose maximum insertion loss can be less than 3 dB. To fill the gas safely and efficiently, two homemade gas cells are applied in two junctions. The 0.2–0.3 MPa pressure difference between the ends of the HC-PCF makes the acetylene gas fill the HC-PCF within 30 s. Meanwhile the residual acetylene gas can be exhausted by filling high-purity nitrogen gas into the HC-PCF in the same way.

**Figure 3:**
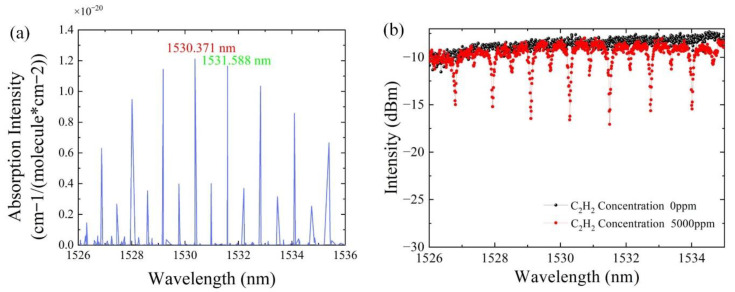
(**a**) Absorption intensity of acetylene in Hytran database, and (**b**) the absorption intensity measured by experiment.

The absorption peak of acetylene from 1526 nm to 1536 nm is shown in Figure 3a [22], and the absorption intensity of acetylene gas measured by experiment is shown in Figure 3b. In previous studies, it can be found that the decrease in filter bandwidth can narrow the linewidth [23], which leads to an increase in sensing sensitivity. According to the ITU grid channels and the position of absorption peak, we chose DWDM filters whose central wavelengths are 1530.33 nm and 1531.9 nm with the spacing of 100 GHZ.

**Figure 4:**
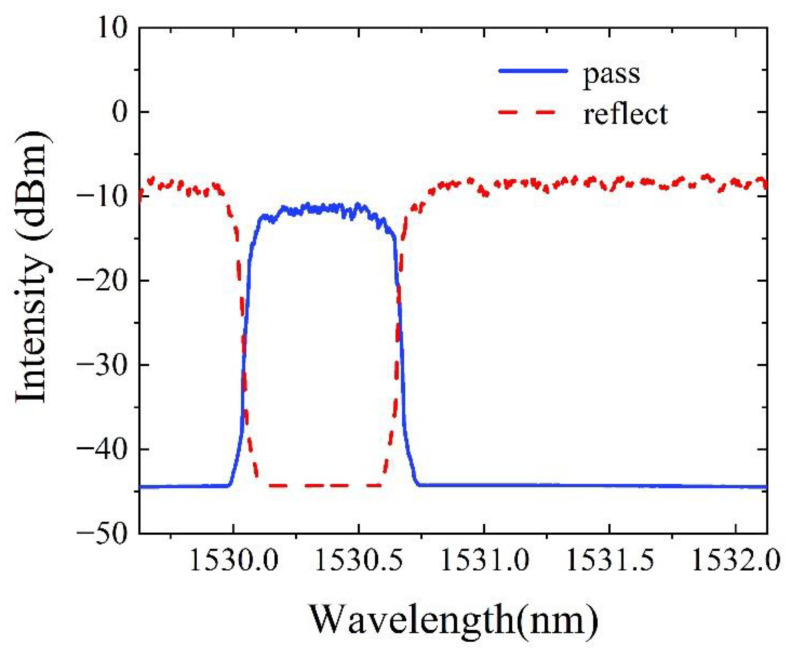
The working principle of the DWDM in the experiment.

The working principle of DWDM in our experiment is shown in Figure 4, which is demonstrated by a scanning wavelength from 1529.13 nm to 1532.13 nm with a step of 20 pm. First, the pass port of DWDM is disconnected, and the spectra during the wavelength range from 1529.13 nm to 1532.13 nm is shown in Figure 4, which is expressed with a red dotted line. Then, the reflect port of DWDM is disconnected, and the spectra is expressed with a solid blue line. As we can see, the DWDM separates the laser output wavelength range into two sections efficiently with an extinction ratio over 30 dB. The absorption peaks of acetylene we selected are 1530.372 nm and 1531.581 nm. They are located in the two flat complementary band-pass regions, which can guarantee the high sensing sensitivity.

As we all know, when a 3 dB coupler is used as a splitter, the input power will not be wasted, but when it is used as a combiner, it will waste half of the input power. Therefore, if 3 dB couplers are used to realize beam splitting and combination, the additional loss will be introduced. However, DWDM is manufactured based on the optical filter principle instead of optical coupler principle, thus it will not introduce additional loss in the system, which makes it more efficient as a combiner. Taking full use of pump light in a multiplexing sensing system is of great importance; thus, in our system, 3 dB couplers and DWDMs are used as splitters and combiners, respectively. It is worth mentioning that we realize two-channel sensing with only one DWDM compared with the reference [16], which can greatly save the cost of the sensor network system.

## 3. Results and Discussions

**Figure 5:**
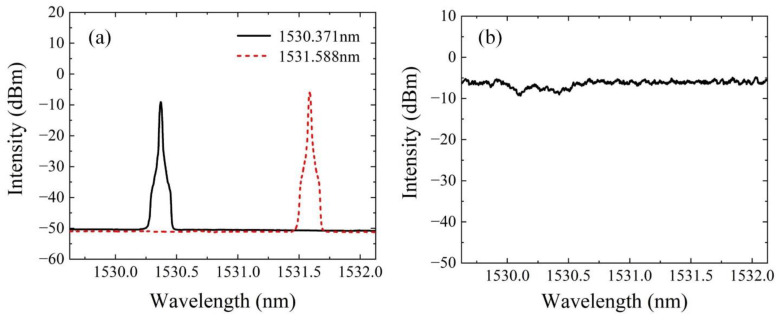
The laser output spectra without acetylene gas.

The output spectra of the laser are shown in Figure 5a, when the scanning wavelengths are 1530.371 nm and 1531.588 nm, corresponding to two absorption peaks of acetylene, respectively. It can be seen that the SNRs of two spectra are above 40 dB and the output intensities of them are close. Then, the scanning wavelength changes from 1529.13 nm to 1532.13 nm with fixed step of 2 pm, when acetylene gas is not filled in the HC-PCFs. The peak values at each wavelength are recorded in Figure 5b. It can be found that the peak values have no obvious fluctuation in the output spectra, but there are two places with lower intensities, which is caused by the filtering characteristics of DWDM. So, as we mentioned, the two sensing wavelengths we selected are located in the flat band-pass region. Then, the laser output scanning spectra after filling acetylene gas will be investigated.

In the gas sensor network system, an optical switch controlled by computer is used to switch different sensing channels in real time, working with an F-P filter applied to scan wavelength containing two absorption peaks synchronously. However, when the optical switch operates continuously, it is a waste of time to wait for the F-P filter to scan the whole band from 1529.13 nm to 1532.13 nm. To enhance the efficiency of the sensor network, the voltage gradient method is adopted. The intracavity absorption multiplexed sensor network can realize the automatic sensor switching by applying a voltage varying linearly to match the wavelength of DWDM to the F-P tunable filter via SMU.

**Figure 6:**
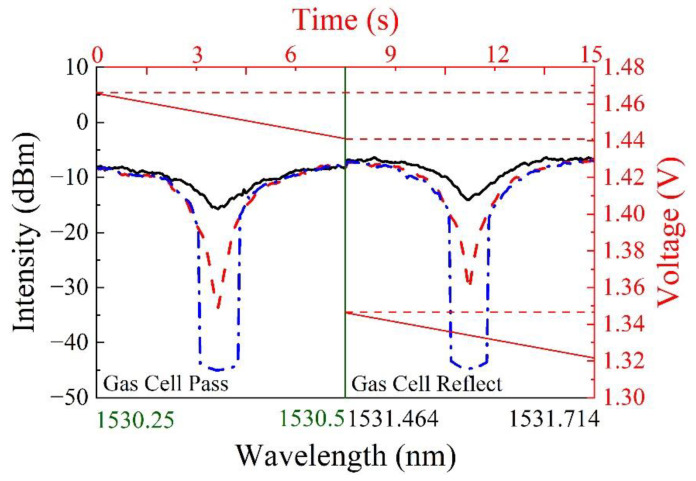
The laser output spectra by using voltage gradient method.

As is shown in Figure 6, the output wavelength of a tunable F-P filter changes linearly with the applied voltage. To compress the scanning time as much as possible, we can only scan the wavelength around the absorption peak by controlling the voltage applied to the tunable F-P filter. When the voltage decreases from 1.466 V to 1.441 V and 1.346 V to 1.321 V, the output wavelength of the tunable F-P filter increases from 1530.25 nm to 1530.50 nm and from 1531.46 to 1531.71, respectively.

Compared with scanning the whole band (from 1529.13 nm to 1532.13 nm), the scanning band width is reduced to one-sixth of the whole band after using the voltage gradient method, and it takes only 15 s to scan the two absorption peaks. That is to say, the voltage gradient method enhances the detection efficiency up to six times.

Then, we expand the two-channel sensor to 16 channels by using a 1 × 8 optical switch. The eight same sensing light paths are connected to the laser cavity in turn by an automatically controlled optical switch. In the experiment, according to the scanning parameters we set, it takes 15 s to complete the scan of one port containing two absorption peaks. As a result, the total time for our sensor network system to scan all 16 channels is 120 s. However, it is worth noting that the scanning time can be shortened significantly in practical application by increasing the scanning step size and narrowing the scanning bandwidth. Furthermore, a high-speed photodiode module and oscilloscope can be used in the system, replacing the OSA, which is the principal factor of limiting scanning speed.

**Figure 7:**
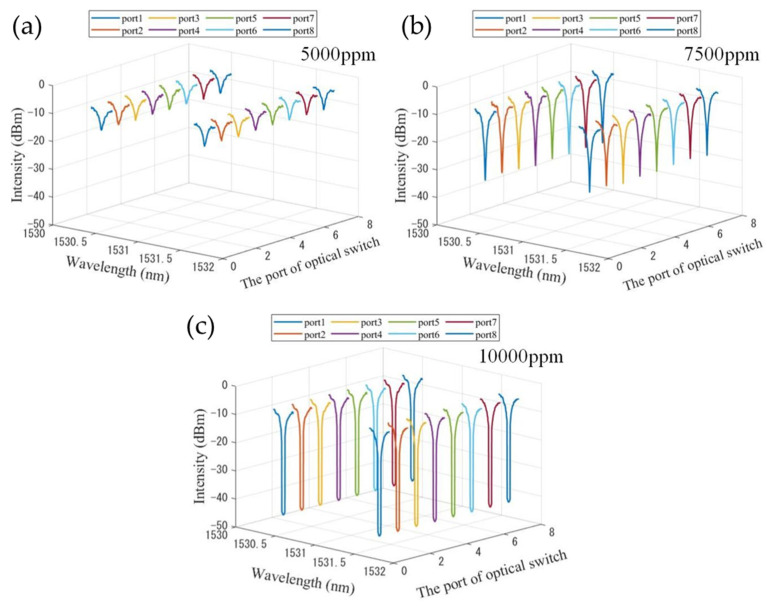
The absorption spectra with different optical switch ports when the acetylene concentration is 5000 ppm, (**a**), 7500 ppm (**b**) and 10,000 ppm (**c**).

When the optical switch is switched to different ports, the absorption spectra with different acetylene concentration is shown in Figure 7. Figure 7a–c show the absorption spectra corresponding to 5000 ppm, 7500 ppm and 10,000 ppm acetylene concentration, respectively. With the voltage gradient method applied in a tunable F-P filter, only the spectra near the two absorption peaks are shown. Eight groups of absorption spectra are obtained at each acetylene concentration. In addition, it can be seen from Figure 7a when the concentration of acetylene is 5000 ppm, the attenuation of the absorption peak is 15 dB. In Figure 7b,c, when the concentration of acetylene is 7500 ppm and 10,000 ppm, the attenuation of the absorption peak is 33 dB and 45 dB, respectively. It can be found that the attenuation of laser output at the absorption peaks increases with the increase in acetylene concentration. This mechanism can be used to retrieve the concentration of acetylene gas.

**Figure 8:**
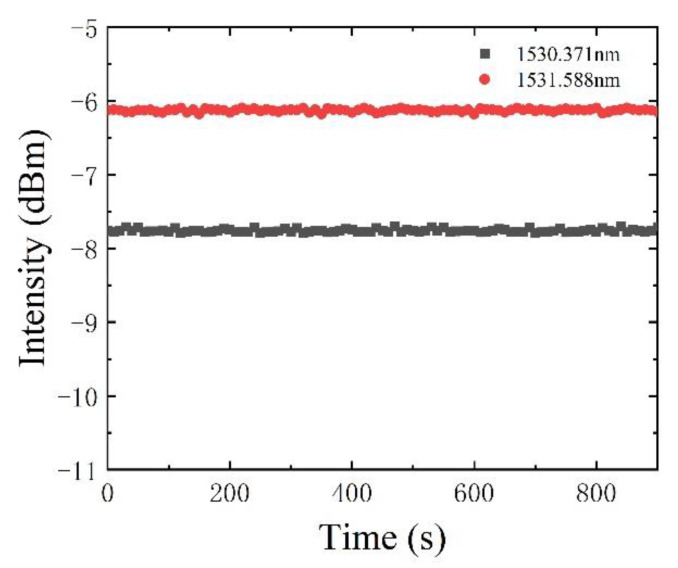
The output power stability of the intracavity absorption gas sensor network.

The power stability is one of the important parameters to measure the quality of a laser, and it is also an important factor to realize accurate detection. We connect one port of the optical switch and fix the output wavelength of the F-P filter at the absorption wavelength over 900 s, as is shown in Figure 8. During this time, the difference between the maximum value and the minimum value of the output intensity is less than 0.1 dBm, which is the fluctuation of the sensing system. The smaller the fluctuation is, the more stable the sensor is. Therefore, the multiplexed intracavity absorption sensor network we proposed has high stability and can satisfy the need for the detection of low-concentration acetylene gas.

The MDL of the sensor network is calculated. In the intracavity absorption sensor, the MDL of the sensor can be expressed as [24]:

(2) $$MDL=\frac{F\times C}{A}$$

where *F* is the fluctuation of the sensor, *C* is the concentration of acetylene gas and *A* is the intensity attenuation at the absorption peak.

In this experiment, we detect three kinds of acetylene concentration: 5000 ppm, 7500 ppm and 10,000 ppm. The MDL at the absorption wavelengths of 1530.371 nm and 1531.588 nm are 25.91 ppm and 26.85 ppm, respectively. Compared with other similar works listed in Table 1, the stability and MDL are improved.

**Figure 9:**
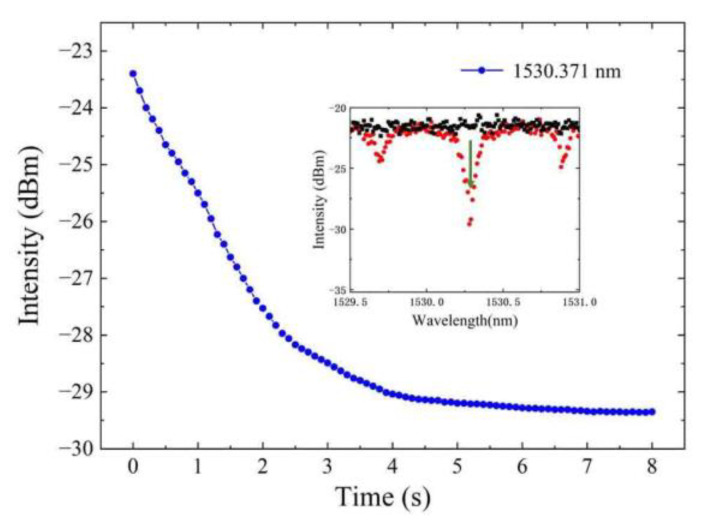
The response time with the pressure difference of 0.5 MPa.

Response time is a very important performance assessment parameter. It is related to detection efficiency. In this paper, the response time was tested. To ensure high accuracy, pure nitrogen is full filled into the gas cell before acetylene filling until there is no absorption line in the spectrum for exhaust. When the filling pressure is 0.5 MPa, the maximum value of absorption loss is reached at 1530.371 nm using about 4 s, as is shown in Figure 9.

## 4. Conclusions

We propose a multiplexed intracavity acetylene gas sensor network combining WDM and TDM and verify its feasibility by an acetylene-gas detection experiment. The optical switch and DWDM are applied in the sensing system at the same time to expand the capacity of the sensor network. The proposed sensing network has 16 sensing points. Meanwhile, the sensing system is optimized through the voltage gradient method, and the detection efficiency of the sensor network is increased up to six times. The experimental results show that the MDL at the absorption wavelengths of 1530.371 nm and 1531.588 nm are 25.91 ppm and 26.85 ppm, respectively. The proposed sensor network has the advantages of high-capacity, high-efficiency, high-stability and low-concentration detection. In addition, the system is compatible with other intracavity sensors, which can realize the detection of many dangerous gases, such as methane, carbon monoxide, ammonia and hydrogen sulfide. Therefore, the sensor network possesses great potential in the detection of low-concentration gas.

## Figures and Tables

**Table 1:** The performance comparison of other similar works.

Characteristics and Ref.	Stability	MDL
Sensor network based on DWDM [16]	<0.5 dB	100 ppm
Sensor based on mode competition [17]	±0.3 dB	398 ppm
Wavelength modulation and wavelength sweep techniques [13]	/	75 ppm
Wavelength sweep technique [15]	/	200 ppm
Mode-locked fiber laser [25]	/	781 ppm
Wavelength modulation technique [12]	/	1000 ppm
Fiber ring laser sensor with 8 m HCPCF [26]	0.2 dB	5.4 ppm
Our work	0.1 dB	25.91 ppm

## Data Availability

Not applicable.
